# The correlation of temporal changes of neutrophil-lymphocyte ratio with seizure severity and the following seizure tendency in patients with epilepsy

**DOI:** 10.3389/fneur.2022.964923

**Published:** 2022-10-20

**Authors:** Hanli Li, Yujing Yang, Mingwei Hu, Xiaoyan Cao, Chuhan Ding, Qibing Sun, Ran Li, Ruonan Liu, Xihai Xu, Yu Wang

**Affiliations:** ^1^Department of Neurology, Epilepsy and Headache Group, The First Affiliated Hospital of Anhui Medical University, Hefei, China; ^2^Department of Health Management Center, The First Affiliated Hospital of Anhui Medical University, Hefei, China

**Keywords:** neutrophil-lymphocyte ratio, inflammation, seizure severity, recurrent seizure attacks, status epilepticus

## Abstract

**Background:**

Changes in the neutrophil-lymphocyte ratio (NLR) has been reported to be associated with epilepsy. Here we aim to investigate the correlation of temporal changes of NLR level with seizure severity and the follow-up seizure attacks in patients with epilepsy (PWE).

**Methods:**

We performed a retrospective analysis of the laboratory data including leukocyte count and NLR within 24 h of acute seizure and during the follow-up period of 5–14 days after acute seizure (NLR1, NLR2, respectively) in 115 PWE, and 98 healthy individuals were included as controls in this study. The correlation of laboratory data with seizure types, etiology of epilepsy, anti-seizure drugs (ASDs), seizure severity, and the follow-up seizure attacks in PWE was studied.

**Results:**

Leukocyte count (*P <* 0.001) and NLR level (*P* < 0.001) were found significantly different between PWE and controls. On the other hand, a multivariable logistic regression analysis showed that NLR1 level (OR = 2.992, *P* = 0.001) and admission leukocyte (OR = 2.307, *P* = 0.002) were both independently associated with acute epileptic seizures. Especially, higher NLR1 level was significantly associated with status epileptics (*P* = 0.013) and recurrent seizures after admission (*P <* 0.001). Furthermore, the multivariable logistic regression analysis indicated that higher NLR1 was a predictor for the tendency of the following recurrent seizure attacks (OR = 1.144, *P* = 0.002). NLR2 was inversely correlated with ASDs taken (*P* = 0.011). Levels of NLR1 (r = 0.441, *P <* 0.001) and NLR2 (r = 0.241, *P* = 0.009) were both positively correlated with seizure severity.

**Conclusions:**

Seizures were correlated with the alterations of systemic inflammation reflected by leukocyte and NLR. NLR1 and admission leukocyte were both independently associated with acute epileptic seizures. Higher NLR1 was associated with status epilepticus and independently predicted the tendency of the following epileptic seizures. NLR2 was significantly associated with ASDs taken. Besides, NLR may be used as a biomarker for seizure severity.

## Introduction

As a chronic neurological disorder, epilepsy affects over 70 million people in the world and imposes a substantial burden on individuals and society (1). It has been found that seizures can significantly impair the quality of human life and this influence depends on the severity of seizures (2). People with epilepsy visit the emergency room more frequently than the general population, with 22% of children and 13% of adults with epilepsy visiting the emergency room each year (3). Acute seizures have long been known to cause higher early mortality in patients with epilepsy (PWE) (4), though a recent report on a more direct and absolute measure of life expectancy has shown that life expectancy is reduced in symptomatic epilepsies, but, prolonged in other subgroups of epilepsies (5). On the other hand, mortality-related sudden unexpected death in epilepsy (SUDEP) can occur immediately during or after a tonic-clonic seizure (6) and it has been shown to be related to the severity of epilepsy (7, 8). Hence, it is necessary to identify possible biomarkers to help predict or mark the development of epilepsy as well as the associated seizure severity for early intervention.

Multiple evidences have indicated the correlation between neuroinflammation and epilepsy. Epileptic seizures provoke neuroinflammation which reciprocally facilitates epileptic seizures (9–12). For example, seizures trigger an increase in pro-inflammatory mediators including COX-2, IL-1β, IL-6, HMGB1, TNF-α and chemokines, which in turn exacerbate epilepsy development (13). Furthermore, neuroinflammation has been shown to contribute to the onset and recurrence of epileptic seizures by lowering its threshold (14). The association of systemic inflammation with epilepsy has also been established (15, 16). Additionally, anti-inflammatory therapy has seizure-suppressing effect (17), such as glucocorticosteroids, an inflammation inhibitor, has significant effect in treating epilepsy of non-inflammatory etiology (18).

Neutrophil–lymphocyte ratio (NLR), which is calculated directly from the complete blood cell count, has been established as a biomarker for systemic inflammation. In previous studies, a relationship between increased level of NLR and central nervous system (CNS) diseases, such as neurodegenerative and cerebrovascular diseases, has been established (19–22). The association of NLR level with epilepsy has been investigated in several studies (23, 24). However, it is still unclear of the relationship of NLR with either the severity of acute seizure or the recurrence of follow-up seizures in PWE. In this study, the association of NLR with the acute seizure severity and the follow-up seizure attacks in PWE will be studied. Besides, the association of temporal changes of NLR level after an acute seizure attack with seizure type, etiology of epilepsy, anti-seizure drugs (ASDs) and status epilepticus (SE) will also be investigated in this study.

## Materials and methods

### Study population

We conducted a retrospective cohort study of PWE in the Department of Neurology, The First Affiliated Hospital of Anhui Medical University from November 2017 to March 2022. This retrospective study was approved by the institutional review board. All PWE were diagnosed according to the International League Against Epilepsy (ILAE) guideline (25). The inclusion and exclusion criteria were shown in Table 1. The inclusion criteria for PWE were: ≥16 years in age, admission for an acute epileptic seizure and availability of a complete blood count with laboratory data at admission (within 24 h of acute seizure) and during the follow-up period of 5–14 days after admission. We excluded patients who had a concurrent or recent infection which was active within 2 weeks before admission including local and systemic infection, such as lung infection, urinary tract infection, and endocarditis. At the same time, acute or subacute traumatic brain injury, acute or subacute cerebrovascular disease within 2 weeks, recent myocardial infarction within 3 months before the study, hematologic diseases, malignancy, and severe liver and kidney diseases were excluded. In addition, patients who were pregnant or receiving immunomodulatory therapy were not included. SE was defined as a seizure lasting ≥30 min, generalized convulsive SE was defined as (1) seizure duration ≥5 min or two or (2) more discrete seizures with incomplete recovery of consciousness between seizures (25). Epilepsy diagnosed for the first time with first prescription of ASDs on admission was defined as newly diagnosed epilepsy (26). Finally, 115 PWE were included in the study.

**Table 1:** The inclusion and exclusion criteria of patients with epilepsy.

**Inclusion criteria**	**Exclusion criteria**
• Diagnosis of epilepsy according to the ILAE criteria; • Age ≥16 years; • Admission for an acute epileptic seizure; • Availability of a complete blood count with differential laboratory data at admission and during follow-up period of 5–14 days after admission.	• A concurrent or recent infection; • An acute or subacute traumatic brain injury; • An acute or subacute of cerebrovascular disease; • A recent myocardial infarction; • Hematological disease; • Malignancy; • Severe liver or kidney disease; • Pregnant; • Receiving immunomodulatory therapy.

PWE, patients with epilepsy.

A total of 500 healthy individuals were selected based on previous health examination records, among which 98 cases were randomly selected as controls. The control group and PWE group were matched in gender and age.

### Data collection and outcome measures

The laboratory data and image information of all patients at admission were retrospectively collected based on the hospital electronic medical record system. Complete blood cell count and leukocyte classification laboratory data were recorded within 24 h of the acute seizure and during a follow-up period of 5–14 days after admission. Blood pressure, C-reactive protein (CRP), liver and kidney function, blood glucose, electrolytes and lipids, electroencephalogram (EEG) and brain magnetic resonance imaging (MRI) were measured in all patients. All brain MRI data were interpreted by experienced neurologists and neuroradiologists who were unaware of the clinical factors of the patients. Duration of illness, epileptic seizure types, the follow-up seizure attacks after admission and the use of ASDs before admission were also collected for all patients. The National Hospital Seizure Severity Scale (NHS3) which contains seven seizure-related factors on a score of 1 to 27 was used to assess the severity of epilepsy (27, 28).

In this study, we reviewed baseline laboratory and clinical factors in patients and healthy controls, including gender, age, systolic blood pressure (SBP), diastolic blood pressure (DBP), albumin, triglyceride (TG) and total cholesterol (TC). A complete blood cell count including total leukocyte count, neutrophil count, and lymphocyte count was collected within 24 h of the acute seizure and during a follow-up period of 5–14 days after admission. As there was no follow-up period in the control group, single laboratory data was included in this study. NLR was calculated by dividing the absolute number of neutrophil by the absolute number of lymphocyte at each time point. NLR at admission within 24 h of an acute seizure was defined as NLR1 and NLR during the follow-up period of 5–14 days after admission defined as NLR2. If more than one laboratory data was available for the NLR, the maximum value was recorded.

### Statistical analyses

All the data were statistically analyzed with SPSS 25.0 statistical software. Continuous variables, if normally distributed, were presented as means ± standard deviation. For non-normally distributed variables, the median of the interquartile range was used for analysis. The independent sample *t*-test and Pearson's correlation were used for comparison of means, the chi-square test was performed for comparison of categorical variables. Mann Whitney U test and Spearman's correlation were used for non-normally distributed data. Univariate and multivariate logistic regression analyses were used to exclude confounding factors and predict risk factors. A receiver operating characteristic (ROC) curve and the area under the curve (AUC) were used to determine the sensitivity and specificity of the NLR diagnostic test. In all analyses, *P* < 0.05 was defined as statistically significant.

## Results

### The main demographic and laboratory characteristics of the PWE group and healthy controls

A total of 115 patients (mean age 45 ± 17 years, age range 16–81 years, 67 male, 48 female) and 98 healthy controls (mean age 45 ± 15 years, age range 18–77 years, 53 male, 45 female) were included in this study. The main demographic and laboratory characteristics of the patients and healthy controls were presented in Table 2. Compared with healthy controls, admission leukocyte count (*P* < 0.001), follow-up leukocyte count (*P* < 0.001), admission neutrophil count (*P* < 0.001), follow-up neutrophil count (*P* < 0.001), levels of NLR1 (*P* < 0.001), NLR2(*P* < 0.001) and TC (*P* < 0.001) were all significantly increased, nevertheless, admission lymphocyte count (*P* < 0.001), follow-up lymphocyte count (*P* < 0.001) and albumin level (*P* < 0.001) significantly decreased in PWE group. No significant differences in gender, age, SBP, DBP and TG were found between two groups.

**Table 2:** Demographic and laboratory characteristics of patients with epilepsy and controls.

	**Epilepsy (*n* = 115)**	**Control (*n* = 98)**	***P* value**
Gender(M / F)	68/47	53/45	0.540
Age (year)	45 ± 17(16–81)	45 ± 15(18–77)	0.969
SBP (mmHg)	126.17 ± 21.56	124.26 ± 14.03	0.910
DBP (mmHg)	77.78 ± 14.41	74.73 ± 9.36	0.134
Albumin (g/L)	40.80(35.50–44.00)	45.85(44.35–47.93)	< 0.001
TG (mmol/L)	1.04(0.79–1.47)	1.27(0.87–1.81)	0.084
TC (mmol/L)	3.97(3.52–4.79)	4.79(4.15–5.35)	< 0.001
Leukocyte1(*10^9^/L)	9.25(6.81–13.10)	5.76(4.81–7.04)	< 0.001
Neutrophil1(*10^9^/L)	6.86(4.23–9.93)	3.18(2.45–4.00)	< 0.001
Lymphocyte1(*10^9^/L)	1.47 (0.96–2.04)	2.02(1.66–2.49)	< 0.001
NLR1	4.41 (2.42–8.65)	1.53(1.23–2.00)	< 0.001
Leukocyte2 (*10^9^/L)	6.50(5.31–7.71)	5.76(4.81–7.04)	< 0.001
Neutrophil2(*10^9^/L)	4.11 (2.86–5.07)	3.18(2.45–4.00)	< 0.001
Lymphocyte2(*10^9^/L)	1.54 (1.21–1.99)	2.02(1.66–2.49)	< 0.001
NLR2	2.40 (1.79–3.44)	1.53(1.23–2.00)	< 0.001
Disease duration (year)	3.89 ± 8.22(0–40)	–	–
NHS3 score	11.15 ± 3.75(1–19)	–	–

M, male; F, female; SBP, systolic blood pressure; DBP, diastolic blood pressure; TG, triglyceride; TC, total cholesterol; Leukocyte1, admission leukocyte count; Neutrophil1, admission neutrophil count; Lymphocyte1, admission lymphocyte count; Leukocyte2, follow–up leukocyte count; Neutrophil2, follow–up neutrophil count; Lymphocyte1, follow–up lymphocyte count; NLR, neutrophil–lymphocyte ratio.

Neutrophil and lymphocyte counts were not included in multi-factor analysis due to the collinearity of NLR with neutrophil and lymphocyte counts. Therefore, univariate analysis showed that the factors associated with seizures were admission leukocyte count, NLR1 level, follow-up leukocyte count, NLR2 level, TC and albumin levels. Multivariate logistic regression analysis (Table 3) showed that NLR1 level (OR = 2.992, *P* = 0.001) was independently associated with acute seizures after adjusting the levels of admission leukocyte, follow-up leukocyte, NLR1, NLR2, TC and albumin. Meanwhile, admission leukocyte count (OR = 2.307, *P* = 0.002) and albumin level (OR = 0.685, *P* < 0.001) were also significantly associated with acute seizures.

**Table 3:** Univariate and multivariate logistic analyses of parameters associated with acute epileptic seizure in patients with epilepsy.

	**Crude OR (95% CI)**	***P* value**	**Adjusted OR (95% CI)**	***P* value**
Leukocyte1	0.559(0.466–0.671)	< 0.001	2.307(1.356–3.923)	0.002
NLR1	0.244(0.153–0.388)	< 0.001	2.992(1.579–5.669)	0.001
Leukocyte2	0.772(0.653–0.913)	0.002	0.652 (0.399–1.064)	0.087
NLR2	0.226(0.137–0.372)	< 0.001	1.395 (0.773–2.517)	0.269
TC	1.763(1.3–2.392)	< 0.001	0.700 (0.424–1.155)	0.162
Albumin	1.398(1.264–1.546)	< 0.001	0.685 (0.576–0.814)	< 0.001

Leukocyte1, admission leukocyte count; Leukocyte2, follow–up leukocyte count; NLR, neutrophil–lymphocyte ratio; TC, total cholesterol.

### Temporal changes of neutrophil-lymphocyte laboratory data for PWE

In PWE group, the numbers of leukocyte (*P* < 0.001) and absolute neutrophil (*P* < 0.001) and the level of NLR (*P* < 0.001) were significantly higher within 24 h of acute seizure compared with the follow-up period of 5–14 days after admission (Table 4).

**Table 4:** Comparisons of laboratory and clinical characteristics between admission and follow–up period in patients with epilepsy.

		**Admission (within 24 h)**	**Follow–up (5–14 days)**	***P* value**
Leukocyte(*10^9^/L)		9.25(6.81–13.10)	6.50(5.31–7.71)	< 0.001
Neutrophil(*10^9^/L)		6.64(4.23–9.84)	4.11 (2.86–5.07)	< 0.001
Lymphocyte(*10^9^/L)		1.47(0.96–2.04)	1.54 (1.21–1.99)	0.201
NLR		4.41 (2.42–8.65)	2.40 (1.79–3.44)	< 0.001
The follow–up seizure attacks	Recurrent seizures	–	33(28.7%)	–
	No seizures	–	82(71.3%)	–
Classification of epilepsy	Newly diagnosed	56(48.7%)	56(48.7%)	–
	Chronic epilepsy	59(51.3%)	59(51.3%)	–
Brain MRI	Abnormal	68(59.1%)	68(59.1%)	–
	Normal	47(40.9%)	47(40.9%)	–
Anti–seizure drugs	Yes	48(41.7%)	48(41.7%)	–
	No	67(58.3%)	67(58.3%)	–
Type of epilepsy	Self–limited seizures	64(55.7%)	64(55.7%)	–
	Status epilepticus	51(44.3%)	51(44.3%)	–

NLR, neutrophil–lymphocyte ratio.

### Analyses of possible predictors of recurrent seizure attacks after admission

**Figure 1:**
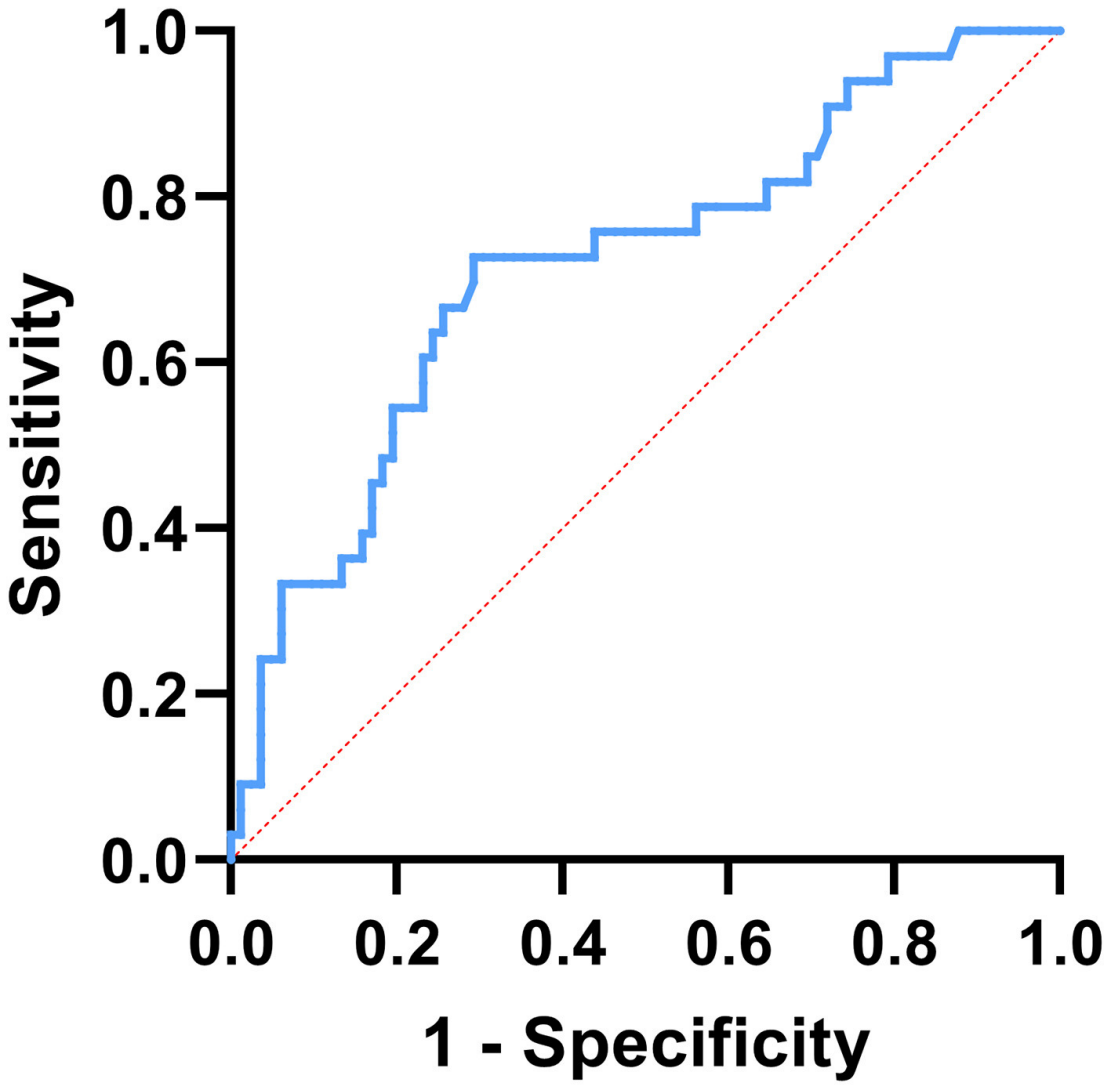
ROC curve analysis of the prediction for the following recurrent seizure attacks based on NLR1. The best cut-off value of NLR1 was 5.56, area under the curve (AUC) = 0.717, *P* < 0.001, 95% CI: 0.611–0.823, sensitivity: 73%, and specificity: 71%.

During the follow-up period of 5–14 days after admission, 33 patients had recurrent seizures before NLR2 level were obtained, and 82 patients had no recurrent seizures (Table 4). Univariate analysis used to identify factors influencing the recurrent seizures after admission showed that higher level of NLR1 and status epilepticus were both associated with the recurrent seizures after admission (Table 5). Furthermore, multivariate logistic regression analysis indicated that NLR1 level was an independent predictor of following recurrent seizures after severe epileptic seizure attacks (OR = 1.144, *P* = 0.002) (Table 6). We further investigated the predictive value of NLR1 for recurrent seizures in these patients after acute seizures. ROC analysis of NLR1 level showed that the best cut-off value was 5.56, AUC was 0.717 (95% CI: 0.611–0.823, *P* < 0.001), and the sensitivity and specificity were 73 and 71% respectively (Figure 1), indicating that the level of NLR1 could predict the risk of recurrent seizures after admission.

**Table 5:** Comparison of baseline characteristics between patients with and without following recurrent seizures after admission.

	**Patients with following recurrent seizures(*n* = 33)**	**Patients without following recurrent seizures (*n* = 82)**	***P* value**
Gender (men)	21	46	0.458
Age (year)	45.27 ± 17.15	45.33 ± 17.60	0.988
Leukocyte 1(*10^9^/*L*)	10.04(7.20–13.71)	8.49(6.19–12.07)	0.101
NLR1	8.01(3.77–14.42)	3.72(2.07–6.70)	0.000
SBP (mmHg)	126.24 ± 18.25	126.15 ± 22.86	0.670
DBP (mmHg)	78.03 ± 14.32	77.68 ± 14.54	0.601
Albumin (g/L)	41.20(35.15–43.80)	40.80(35.63–44.10)	0.819
TG (mmol/L)	1.22(0.84–1.62)	1.00(0.78–1.39)	0.131
TC (mmol/L)	4.31(3.54–5.08)	3.85(3.52–4.59)	0.246
NHS3	11.97 ± 4.03	10.82 ± 3.61	0.137
Chronic epilepsy	15	44	0.426
Abnormal brain MRI	20	48	0.838
SE	20	31	0.026
ASDs taken	14	34	0.925
Disease duration (≥5 years)	10	13	0.080

Leukocyte1, admission leukocyte count; NLR, neutrophil–lymphocyte ratio; SBP, systolic blood pressure; DBP, diastolic blood pressure; TG, triglyceride; TC, total cholesterol; SE, status epilepticus.

**Table 6:** Univariate and multivariate logistic analyses of possible predictors for the following recurrent seizure attacks after admission.

	**Crude OR (95% CI)**	***P* value**	**Adjusted OR (95% CI)**	***P* value**
NLR1	1.156(1.066–1.253)	0.000	1.144(1.053–1.243)	0.002
SE	2.531(1.105–5.797)	0.028	0.523(0.216–1.266)	0.151

NLR, neutrophil–lymphocyte ratio; SE, status epilepticus.

### The correlation between NLR1 and NLR2 in PWE

**Figure 2:**
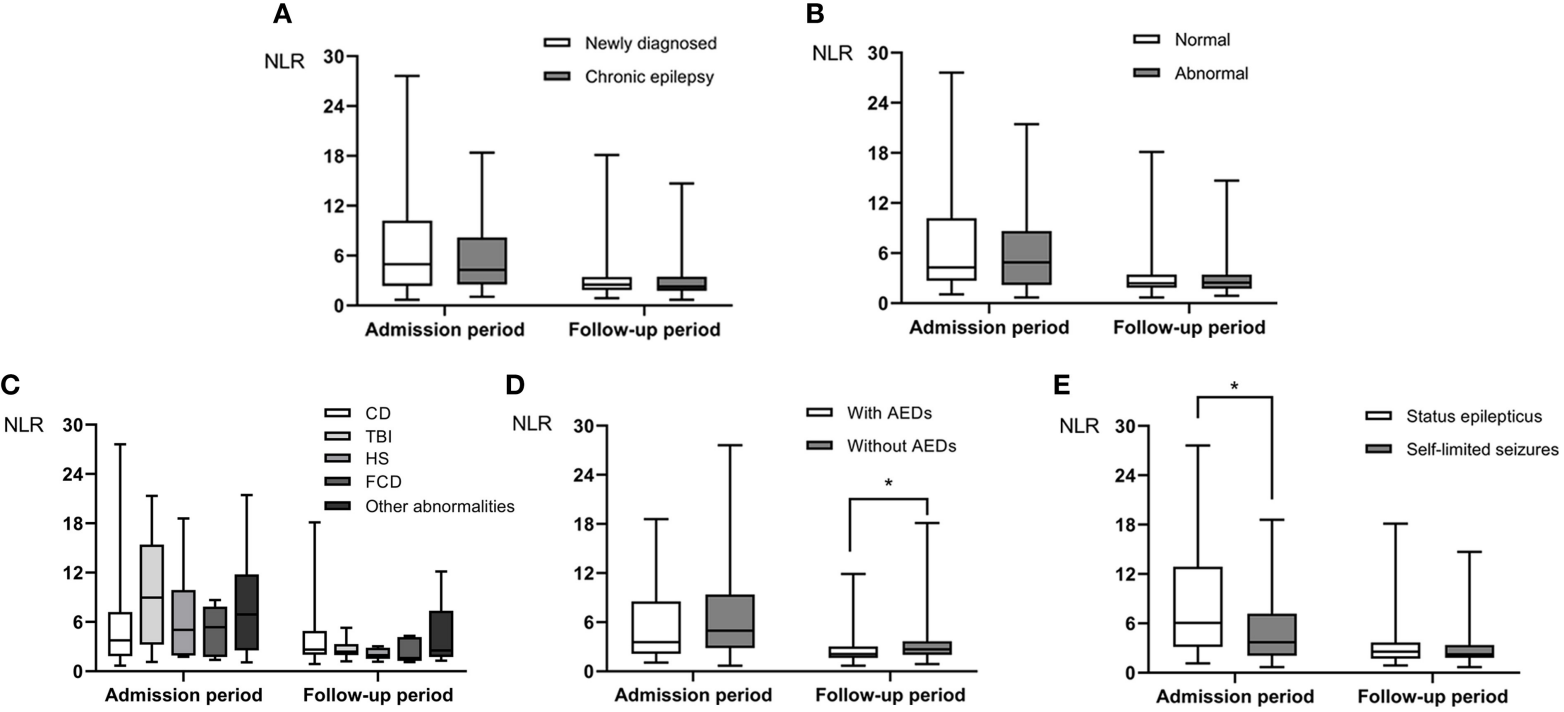
The difference in NLR levels at admission (NLR1, within 24 h of acute seizure) and follow-up period (NLR2, 5–14 days after admission) in PWE. Neither NLR1 nor NLR2 level was different between patients with newly diagnosed epilepsy and those with chronic epilepsy **(A)**. There was no significant difference in NLR level between patients with idiopathic epilepsy and those with secondary epilepsy **(B)**, and there was no significant difference in NLR level among the five categories of patients with focal epilepsy **(C)**. NLR2 level in patients on treatment with ASDs was lower than those in patients on no treatment with ASDs **(D)**. NLR1 level in patients with SE was higher than that in patients with self-limited seizures **(E)**. Group comparison was done by Mann-Whitney U test. *P < 0.05.

Sixteen out of 115 patients (13.9%) had focal seizures and 9 of the 16 patients also had secondary bilateral tonic-clonic seizures. Ninety nine out of 115 (86.1%) had generalized seizures and 84 of the 99 patients had generalized tonic-clonic seizures. Out of the 115 patients, 56 had newly diagnosed epilepsy and 59 had chronic epilepsy. Abnormal brain MRI was found in 68 patients and normal brain MRI found in 47 patients. Fifty one patients were admitted because of SE and the remaining 64 patients admitted because of self-limited seizures. Before admission, 48 patients were on treatment with ASDs and the others were not on treatment with ASDs. Regarding to the NLR levels measured within 24 h of acute seizure and during the follow-up period of 5–14 days after admission, no difference was found in NLR level between newly diagnosed epilepsy and chronic epilepsy (NLR1: *P* = 0.546, NLR2: *P* = 0.474) (Figure 2A, Table 4). Also, no significant difference in NLR level was found between patients with and without abnormal brain MRI (NLR1: *P* = 0.712, NLR2: *P* = 0.833) (Figure 2B, Table 4). To investigate the effect of different focal lesions on the level of NLR, 68 patients with abnormal brain MRI were divided into subgroups secondary to cerebrovascular disease (CD) (31 cases), traumatic brain injury (TBI) (14 cases), hippocampal sclerosis (HS) (6 cases), focal cortical dysplasia (FCD) (5 cases), and subgroup secondary to other abnormalities (12 cases). No significant difference in NLR level was found among the five categories of patients with focal seizures (NLR1: *P* = 0.368, NLR2: *P* = 0.536) (Figure 2C). As for the treatment with ASDs before admission, 26 cases were treated with monotherapy and 22 treated with combined drugs. As for types of ASDs, sodium valproate was used in 25 cases, carbamazepine in 12 cases, levetiracetam in 10 cases, lamotrigine in 9 cases, and other ASDs including phenobarbital, topiramate and etc. in the remaining cases. With regard to NLR1 level, no difference was found between patients who received treatment with ASDs and patients who did not (*P* = 0.370). But, for NLR2 level, it was significantly lower in patients who received treatment with ASDs (Figure 2D, Table 4) than that in patients did not (*P* = 0.011). Finally, NLR1 level was found significantly higher (Figure 2E, Table 4) in patients admitted because of SE than that in patients admitted because of self-limited seizures (*P* = 0.013), but no difference of NLR2 levels was found between these two groups of patients (*P* = 0.290) (Figure 2E, Table 4).

### The correlations between NLR level and seizure severity

**Figure 3:**
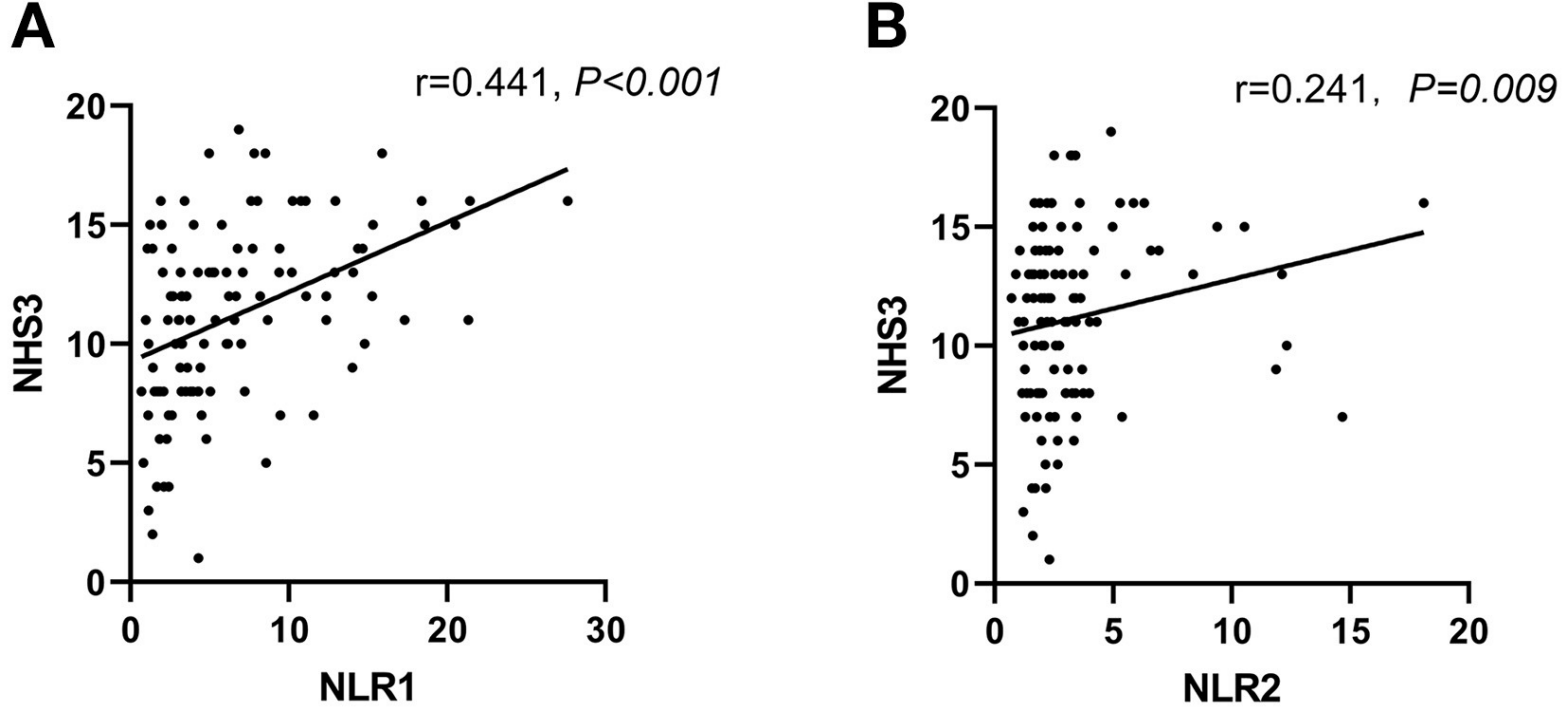
Levels of NLR were positively correlated to NHS3 scores in PWE at admission (within 24h of acute seizure) **(A)** and follow-up period (5–14 days after admission) **(B)**. Correlation analysis was done by Spearman's correlation.

Seizure severity was assessed by NHS3 scale scores in this study. To evaluate the correlation between NLR level and seizure severity, we found that NLR level was positively correlated with NHS3 scores at admission and during follow-up (Spearman's correlation r = 0.441, *P* < 0.001, and r = 0.221, *P* = 0.009, respectively) (Figures 3A,B).

## Discussions

In this retrospective study, 115 patients included were all hospitalized because of acute seizures. Among the 115 patients, 51 patients (44.3%) were admitted because of SE and 56 admitted because of newly diagnosed epilepsy. Forty eight patients were accepting treatment with ASDs before admission. The type of seizures was mainly generalized tonic-clonic seizures.

Studies have indicated an involvement of leukocyte in the pathophysiology of epilepsy (16, 29, 30). The function of peripheral immune cell subsets is altered during epileptic seizures, such as upregulation of CD32-expressed granulocytes and monocytes reduction of HLA-DR-expressed monocytes after seizures (30), and this monocyte infiltration may lead to the recurrence of epileptic seizures (31). On the other hand, a significant increase in the number of blood leukocytes after seizures has been reported in patients with generalized or focal onset seizures, with peripheral blood leukocyte counts above the upper limit of normal in approximately one-third of patients after generalized onset seizures (16, 32). In this retrospective cohort study, we demonstrated that, compared to healthy controls, PWE had higher levels of leukocyte and NLR at admission within 24 h of acute seizure and during the follow-up period. Furthermore, multivariate logistic regression analysis illustrated that the levels of admission leukocyte and NLR1 were both independently associated with acute epileptic seizures. This indicated that inflammation reflected by NLR was involved in the pathophysiology of epilepsy. It is reported that serum albumin can extravasate from blood vessel into the brain parenchyma when the blood-brain barrier is dysfunctional, and this has been suggested to be involved in the pathogenesis of various types of epilepsy (33–35). In this study, we found that low serum albumin was associated with acute seizures, which effectively confirmed albumin extravasation after acute seizures.

Meanwhile, NLR1 level was found to be a significant predictor for recurrent epileptic seizures after admission in this study. According to the ROC analysis for NLR1, we found that the best cut-off value was 5.56 and the AUC was 0.717 (95% CI: 0.611–0.823, *P* < 0.001) with a sensitivity of 73% and a specificity of 71%. This finding suggested that an elevated NLR shortly after an acute seizure may predict a tendency for recurrent seizures in the following days, which indicates that the inflammation reflected by NLR may play a causative role in epileptogenesis. Multiple studies have shown that there is a reciprocal interaction between inflammation and epilepsy (9, 10, 13). Epileptic seizures can disrupt the blood-brain barrier and activate a variety of cells including microglia, astrocytes, mononuclear macrophages and neutrophil in the CNS. Subsequently, the activated microglia, reactive astrocytes and infiltrating immune cells release large amounts of pro-inflammatory mediators including IL-6, IL-1β, TNF-α, COX-2, HMGB1 and chemokines, inducing neuroinflammation through multiple signaling pathways. And the neuroinflammation can further increase excitability of the CNS, along with serum albumin extravasation (14, 35, 36). Thus, neuroinflammation can aggravate the severity, duration, and frequency of epileptic seizures and even lead to new-onset seizures (13, 15, 37). On the other hand, patients with systemic autoimmune diseases, including systemic lupus erythematosus (SLE) and Hashimoto's thyroiditis, especially children have a significantly increased risk of epilepsy (38). These results suggest that epilepsy is associated with systemic inflammation that can lead to abnormal neuronal connection and neuronal hyperexcitability, thereby mediating the development of epilepsy (10, 17).

In this study, we also found that NLR and leukocyte levels were higher within 24 h of acute seizure than during the follow-up period of 5–14 days after admission. Consistent with previous studies, the increase in NLR level induced by acute seizures subsequently decreased (24), but NLR level remained higher than normal controls during the follow-up period of 5–14 days after admission. From the literature review, most of the previous studies concerning the relationship between epilepsy and NLR were conducted during the acute and subacute stages of seizures, with a maximum follow-up time of 96 h (23, 24). This study is the first to investigate temporal changes in NLR levels during a follow-up period of 5–14 days after an acute epileptic seizure. We found that systemic inflammatory responses reflected by NLR remained increased during 5–14 days of follow-up after acute seizures compared with healthy controls. Some studies have also found that NLR level is higher in patients with chronic temporal lobe epilepsy than in healthy controls (39). Accordingly, it is suggested that the long-lasting systemic inflammatory response induced by acute seizures may be involved in the process of epileptogenesis either in the acute stages or in the chronic stages.

Our study found that there was no difference in NLR levels between the group of patients with newly diagnosed epilepsy and the group of patients with chronic epilepsy, nor between the group of patients with normal brain MRI and the group of patients with focal seizures caused by CD, TBI, HS, FCD and other lesions. This may indicate that the seizure-induced inflammatory response reflected by blood NLR levels is similar in different epileptic causative conditions. Whereas, it should be noted that several studies have shown that the levels of inflammatory cytokines vary with the etiology and historical duration of epilepsy (11, 40). The reason for this inconsistency may be the different inclusion criteria of our study subjects, or the different of our study to reflect inflammatory response compared with literature reports. Thus, our results suggest that, at least in part, NLR levels depend neither on the simple cumulative effect of recurrent seizures nor on the etiology of epilepsy.

In this study, most patients were already on treatment with ASDs before admission, which mainly included valproic acid, lamotrigine, levetiracetam and carbamazepine. We found that NLR2 level, i.e., NLR level during the follow-up period of 5–14 days after admission, was lower in patients on medications with ASDs than that in patients not on medications with ASDs. On the other hand, previous studies had demonstrated the anti-inflammatory effects of ASDs in epilepsy treatment (41–44). Therefore, we speculate that ASDs may effectively promote the return of NLR to baseline, thereby facilitating the recovery of acute seizure-induced inflammation.

SE is an acute and potentially life-threatening emergency with high morbidity and mortality (45). Proinflammatory events in the periphery or brain have been shown to govern SE occurrence, and SE-induced neurological dysfunction and even death are significantly associated with SE duration (37, 46). In this study, we found that NLR1 level was higher in patients admitted with SE than that in patients admitted with self-limited seizures, implicating that SE induces more severe inflammatory response compared with self-limited seizures. This is consistent with a report that half of the patients developed systemic inflammatory response syndrome (SIRS) after SE, which is considered to be an independent risk factor for drug resistance and death (47). Therefore, routine inflammation assessment, especially NLR assessment, should be performed in SE patients to assess the prognosis of epilepsy and intervene as early as possible.

For patients with uncontrolled epilepsy, seizure severity may be more important than seizure frequency (48). The severity of epilepsy can be assessed by various scales in clinical practice. In this study, NHS3, a valid and easy-to-apply measure, was used to quantitatively assess epilepsy severity (27, 28). We found a significant correlation between NLR levels and NHS3 scores not only within 24 h of acute seizure but also during the follow-up period of 5–14 days after admission. Our study is the first to demonstrate a correlation between NLR level and epilepsy severity, suggesting that NLR is a biomarker for seizure severity. Patients with epilepsy often experience recurrent seizures and generalized tonic-clonic seizures greatly increase the risk of SUDEP depending on the severity of epilepsy (6, 49). Together with the findings that NLR1 level was independently associated with acute seizures and an elevated NLR shortly after an acute seizure may predict a tendency of recurrent seizures in the following days, we believe that monitoring of NLR level can help to assess the severity of acute attacks, so as to identify patients at high risk for developing subsequent seizures and implement effective interventions early to reduce morbidity and mortality.

This study has the following limitations. First, because of its retrospective design, a large number of patients without NLR data were excluded. Second, we recorded NLR2 from a single measure over 5–14 days because the sample size was not large enough. The dynamic changes of neutrophils, lymphocytes and NLR levels can be evaluated more accurately with a larger sample size in the future.

## Conclusions

In conclusion, we found that epileptic seizures were associated with inflammatory responses reflected by leukocyte and NLR levels. NLR1 level was independently associated with acute epileptic seizures. Meanwhile, NLR1 level was an important predictor of seizure recurrence after admission. Therefore, NLR-reflected inflammatory responses is a consequence of seizures and may also be responsible for the development of epilepsy. Furthermore, NLR1 and NLR2 levels were respectively associated with SE and use of ASDs, and there was a positive correlation between NLR level and seizure severity, indicating that NLR may be used as a biomarker for seizure severity. We suggest that monitoring of NLR levels should be performed as early as possible to help predict the risk of seizure recurrence following acute epileptic seizure attack and intervene as early as possible.

## Data availability statement

The original contributions presented in the study are included in the article/supplementary material, further inquiries can be directed to the corresponding authors.

## Ethics statement

The studies involving human participants were reviewed and approved by Ethics Committee of The First Affiliated Hospital of Anhui Medical University. Written informed consent from the participants' legal guardian/next of kin was not required to participate in this study in accordance with the National Legislation and the Institutional Requirements.

## Author contributions

HL explained the data and wrote the paper. YY, MH, and XC acquired and analyzed data. CD, QS, and RLi performed the literature search and data collection. RLiu and XX contributed data curation and investigation. HL and YW designed the study and revised the manuscript. All authors approved the final manuscript.

## Funding

This study was financially supported by the National Natural Science Foundation of China (YW, Grant No. 82071460).

## Conflict of interest

The authors declare that the research was conducted in the absence of any commercial or financial relationships that could be construed as a potential conflict of interest.

## Publisher's note

All claims expressed in this article are solely those of the authors and do not necessarily represent those of their affiliated organizations, or those of the publisher, the editors and the reviewers. Any product that may be evaluated in this article, or claim that may be made by its manufacturer, is not guaranteed or endorsed by the publisher.
